# Editorial: Animal models of neuropsychiatric disorders: validity, strengths, and limitations

**DOI:** 10.3389/fnbeh.2023.1200068

**Published:** 2023-05-12

**Authors:** Thibaut Sesia, Jennifer M. Wenzel, Boriss Sagalajev, Ali Jahanshahi, Veerle Visser-Vandewalle

**Affiliations:** ^1^Department of Stereotactic and Functional Neurosurgery, Faculty of Medicine and University Hospital Cologne, University of Cologne, Cologne, Germany; ^2^European Graduate School of Neuroscience (EURON), Maastricht, Netherlands; ^3^Department of Psychological Sciences, University of San Diego, San Diego, CA, United States; ^4^Department of Neurosurgery, Maastricht University Medical Center, Maastricht, Netherlands

**Keywords:** animal model, criteria of validity, neuropsychiatric disorders, editorial, Research Topic

The COVID-19 global pandemic brought attention to the continuous debate about the value and necessity of animal testing, as researchers sought to develop a vaccine as quickly as possible. Animal models typically are the first source of vaccine development; however, due to time pressure imposed by the pandemic, some biotech companies used alternative development platforms, and their developed vaccines were later approved by multiple federal agencies for human testing without validation in animals. This led to questions of whether animal modeling is necessary to develop safe and effective therapies. However, this line of questioning ignores the decades of animal testing required to validate vaccine components and assumes that the value of animal models lies in safety testing alone. In this Research Topic, we highlight the strengths and limitations of animal models of neuropsychiatric disorders and provide evidence that they remain an invaluable tool in the ever-expanding arsenal of scientific methods.

Animal models as scientific tools are sophisticated, constantly evolving, and more powerful than ever, and so are the systems to evaluate their translational relevance and rigor. Theoretical frameworks around animal modeling have been proposed since 1960, with Willner's approach on the validity of animal models for depression being arguably the most referenced (Willner, 1984). In Willner's work, there are three dominant criteria: (1) face validity (resemblance to the human disease), (2) construct validity (similar etiology to the human disease), and (3) predictive validity (ability to predict therapeutic effectiveness, or lack thereof, of treatments in the human disease). Belzung and Lemoine (2011) offer a recent update that incorporates new technical advances and updates in scientific knowledge (Belzung and Lemoine, 2011). They develop their framework around the life course of the organism, emphasizing a need for criteria of validity to be met at each pivotal transition that brings an organism from a healthy state to pathological and convalesced. This enhanced set of criteria results in a more representative, flexible, and widely applicable framework. Their criteria of validity highlight how the articles selected for this Research Topic can help form a broader and more informed opinion on the purpose of animal models in research on neuropsychiatric disorders.

From an experimental standpoint, modeling behaviors related to human neuropsychiatric disorders in animals is a complex task given that many central symptoms of human neurobiological diseases do not culminate in behaviors typical of animals, there are a lack of biomarkers and objective diagnostic tests for many neuropsychiatric diseases, and the heterogeneity of symptomatology brings to question if the underlying neurobiology of the disease varies among patients. The work of McNaughton makes the compelling case that producing superior animal models necessitates robust neuropsychological theories to lean on. The relationship between brain networks, symptoms, and effect-based drug actions in anxiolytics is discussed.

One of the expectations for some animal models is to provide a highly predictive tool to test putative new therapies, particularly drugs. The identification of potential new antidepressants, for instance, strongly relies upon drug effects on behavior during the forced swim test in rodents. Since its inception, the forced swim test has been modified several times; Rosas-Sanchez et al. discuss beneficial and deleterious changes brought to the task to bring to light limitations of this model and discuss how to move forward.

In addition, animal models are powerful tools to examine etiological underpinnings of complex disorders. For instance, animal models can elucidate early environmental and triggering factors that lead to disease development in vulnerable organisms, which are nearly impossible to pinpoint in human models given the complex and high-noise environment of typical human life. In this Research Topic, Ferraro et al. establish a link between in-utero exposure to valproic acid, a model long used to induce autism spectrum disorder (ASD)-related behaviors in rodents, and alterations in clock-gene expression. Their data provides insight into the neurobiological mechanisms underlying circadian regulation and ASD, a disease which is marked by sleep-wake cycle disturbances. Additionally, Lopez-Moroga et al. review the effects of acute and chronic stress and active and passive avoidance behavior, a hallmark of several psychiatric disorders, and discuss how variations in the behavioral model used may contribute to the disparate findings reported in the literature.

Finally, neuropsychiatric disorders are composed of multiple symptoms that are not all present in every patient, and sometimes even at odds with each other. A value of animal models is the ability to recapitulate a single behavioral symptom of an otherwise convoluted syndrome in isolation to investigate its neurobiological mechanisms. Here Waku et al. present a novel model of paradoxical kinesia to show that exposure to aversive stimuli can reverse haloperidol-induced catalepsy in rats. This work contributes to a growing body of evidence suggesting interactions between systems subserving emotion and movement, and sets forward an animal model for investigating neural mechanisms underlying how emotional stimuli may influence motor function in Parkinson's patients.

In summary, this Research Topic highlights the strengths and limitations of animal models in neuropsychiatric research. It proposes that animal models remain invaluable, powerful tools uniquely suited to identify theoretical mechanisms and address practical challenges. However, as discussed by Uliana et al. animal models cannot be expected to exactly recapitulate complex human neuropsychiatric syndromes. Thus, animal models must be carefully selected by researchers to closely model specific symptoms of human disease in order to dissect underlying neurobiology, while carefully considering criteria of validity such as those as described by Willner (1984) and Belzung and Lemoine (2011).

## Author contributions

TS, JW, BS, AJ, and VV-V participated in editing the manuscript and agree to be accountable for the content of the work. All authors contributed to the article and approved the submitted version.
